# An odontogenic cutaneous sinus tract in an unusual site with multiple misdiagnoses

**DOI:** 10.1002/ccr3.8049

**Published:** 2023-10-15

**Authors:** Pegah Sarraf, Mehrfam Khoshkhounejad, Maryam Babaahmadi

**Affiliations:** ^1^ Department of Endodontics, School of Dentistry Tehran University of Medical Sciences Tehran Iran; ^2^ Dental Research Center, Dentistry Research Institute Tehran University of Medical Sciences Tehran Iran

**Keywords:** cutaneous sinus tract, misdiagnosis, odontogenic sinus tract, root canal therapy

## Abstract

**Key Clinical Message:**

Dental clinicians and physicians should be careful in differential diagnosis of facial cutaneous nodules, since they might have an odontogenic origin.

**Abstract:**

Odontogenic cutaneous sinus tracts are commonly misdiagnosed and mismanaged; thus, they are prone to recurrence. Herein, a 21‐year‐old female patient is reported with a red fluctuant nodule on her right cheek which had been misdiagnosed as an epidermoid cyst, cystic acne, and parotid gland fistula. The odontogenic origin of the lesion was first suspected when the patient presented to the Department of Endodontics, Faculty of Dentistry for a routine dental check‐up. Multiple‐visit non‐surgical root canal retreatment of the maxillary right first molar, without any additional treatment, resulted in shrinkage of the lesion. After 1 year, the lesion was resolved completely, the respective tooth and the cheek were asymptomatic, and the patient had no complaint.

## Graphical abstract

Healing of a cutaneous sinus tract associated with maxillary first molar after non‐surgical root canal retreatment.

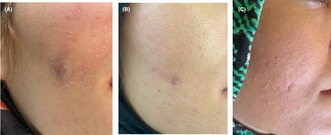

## 1 INTRODUCTION

Odontogenic cutaneous sinus tracts are amongst the skin lesions that are often misdiagnosed due to their rare occurrence and absence of dental signs/symptoms.^1^, ^2^ They are nodulocystic lesions occurring most frequently on the chin or jaw.^3^ Patients with such lesions often undergo multiple surgical excisions, radiotherapy, multiple biopsies, and different antibiotic therapy protocols. However, since such treatments do not eliminate the odontogenic origin of the lesion, the sinus tract continues to recur.^2^ Atypical locations of such lesions may lead to their misdiagnosis, unnecessary treatments, and persistence.^1^ In such cases, root canal treatment or tooth extraction is the definite treatment to remove the main source of infection. Herein, we present a distant cutaneous sinus tract misdiagnosed as an epidermoid cyst, cystic acne, and salivary gland fistula. Previous reports of odontogenic cutaneous sinus tracts associated with a maxillary tooth are available; however, to the best of the authors' knowledge, the present case is the second reported case of a cutaneous sinus tract associated with a maxillary first molar tooth.

## 2 CASE PRESENTATION

**FIGURE 1:**
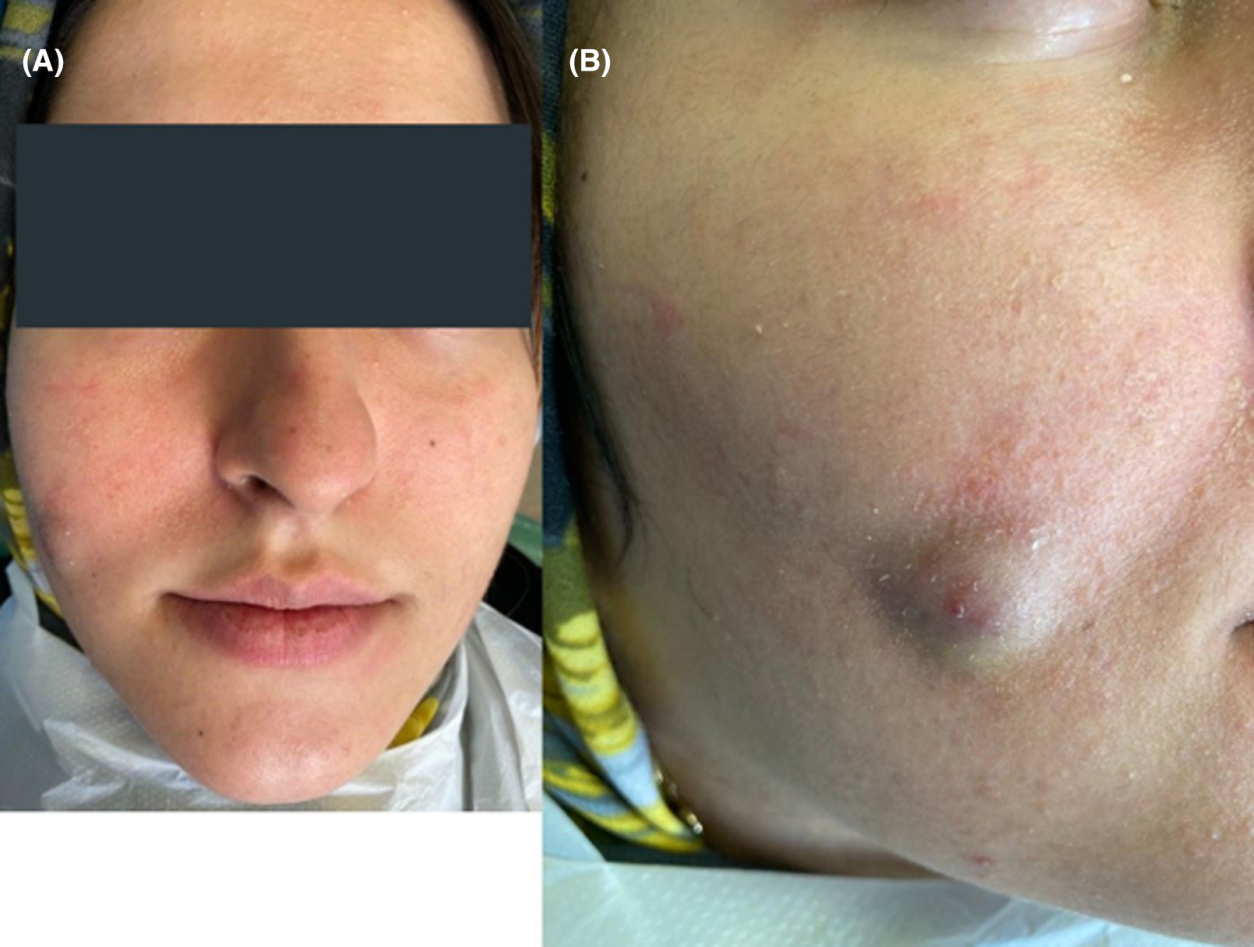
Clinical images of the nodular lesion on patient's right cheek (A) and (B).

A 21‐year‐old female patient presented with a 1‐cm tender red fluctuant nodule on her right cheek that first appeared about a year ago (Figure 1). She was visited by a dermatologist who prescribed oral azithromycin and intralesional triamcinolone injection, but the lesion was not resolved. Another physician misdiagnosed it as an epidermoid cyst, and recommended surgical excision of the lesion which was refused by the patient. An oral medicine specialist requested ultrasound imaging under the suspicion of a parotid gland fistula. In the meanwhile, the patient presented to the Department of Endodontics for a routine dental check‐up.

**FIGURE 2:**
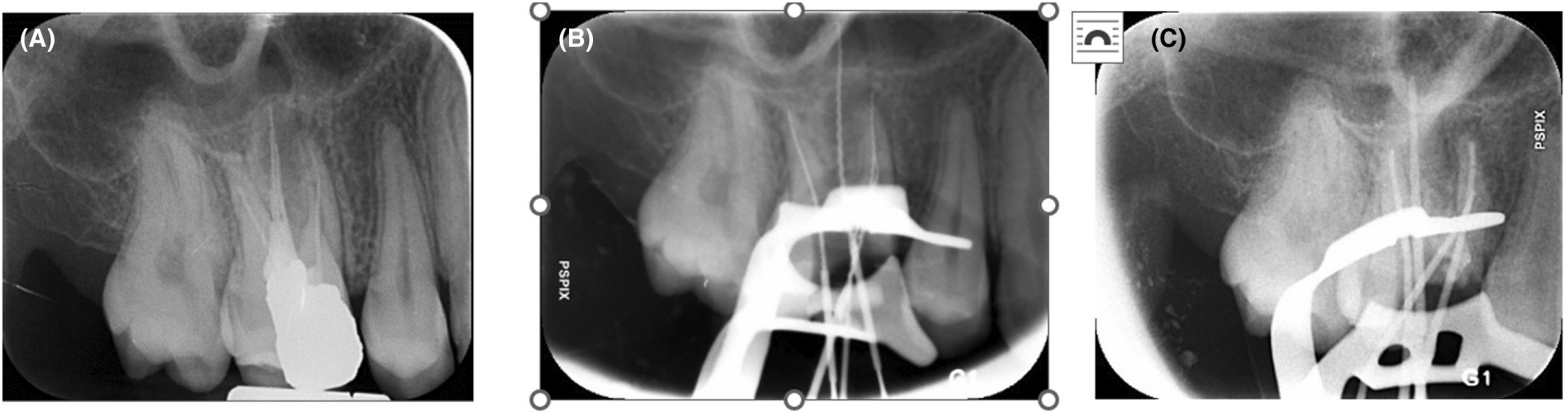
Periapical radiographs; diagnostic (A), initial file (B), cone fit (C).

**FIGURE 3:**
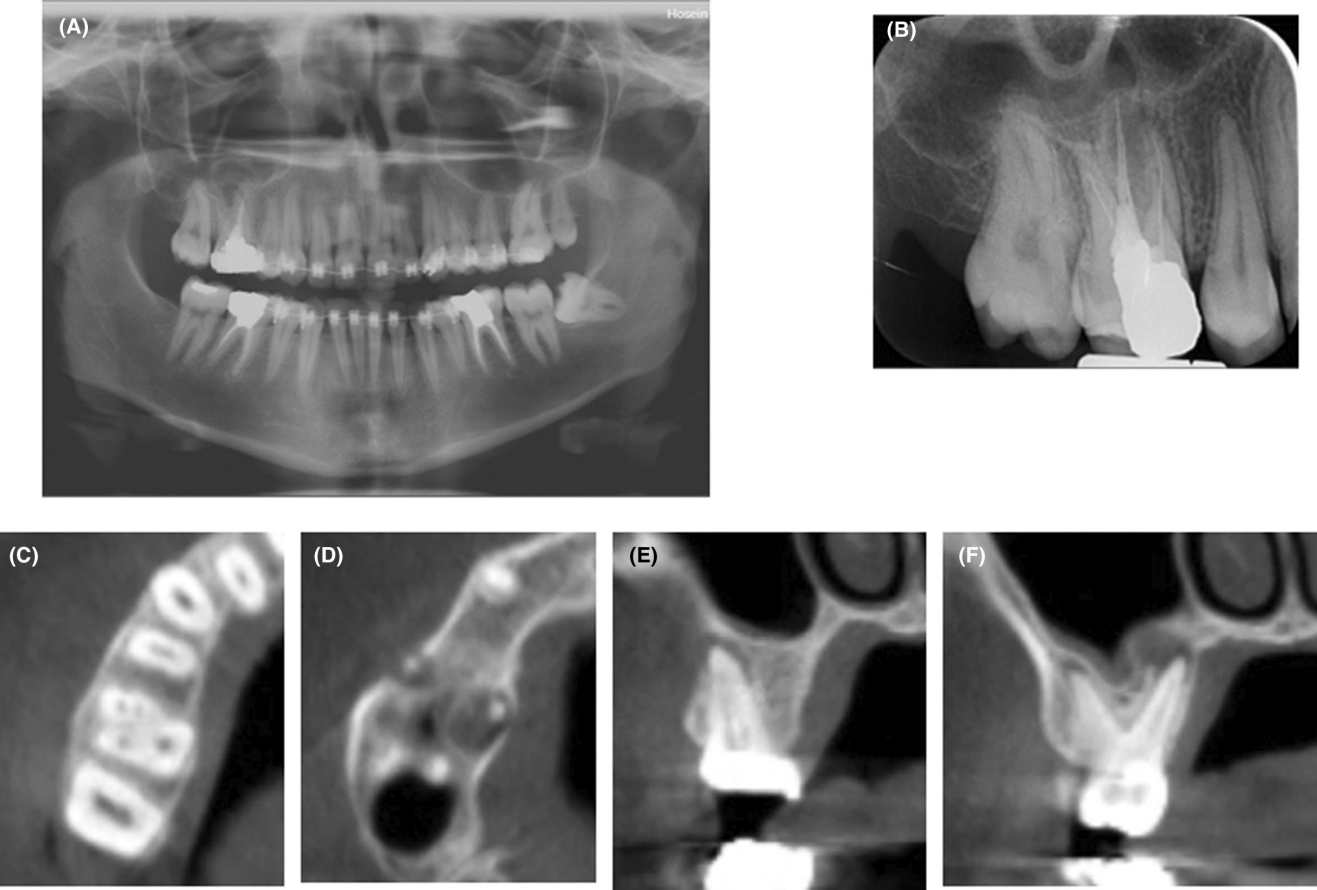
Panoramic view (A), diagnostic periapical radiograph (B), CBCT axial view of right maxillary first molar after the first session of retreatment (C), CBCT axial view showing periapical lesions related to the mesiobuccal and palatal roots; buccal cortex associated with mesiobuccal root apex is perforated (D), CBCT coronal view of mesiobuccal root associated with buccal cortex perforation (E), and CBCT coronal view of palatal and distobuccal roots, Note the mucosal thickening in the right maxillary sinus (F).

**FIGURE 4:**
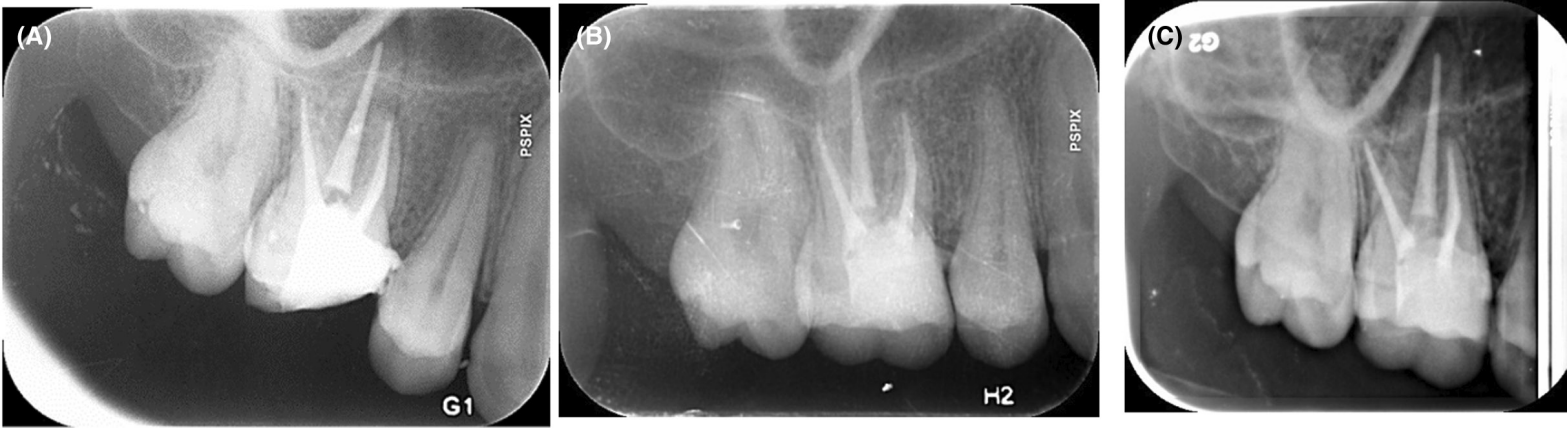
Post‐op periapical radiograph (A), periapical radiograph of 4‐month recall (B), and 12‐month recall (C).

**FIGURE 5:**
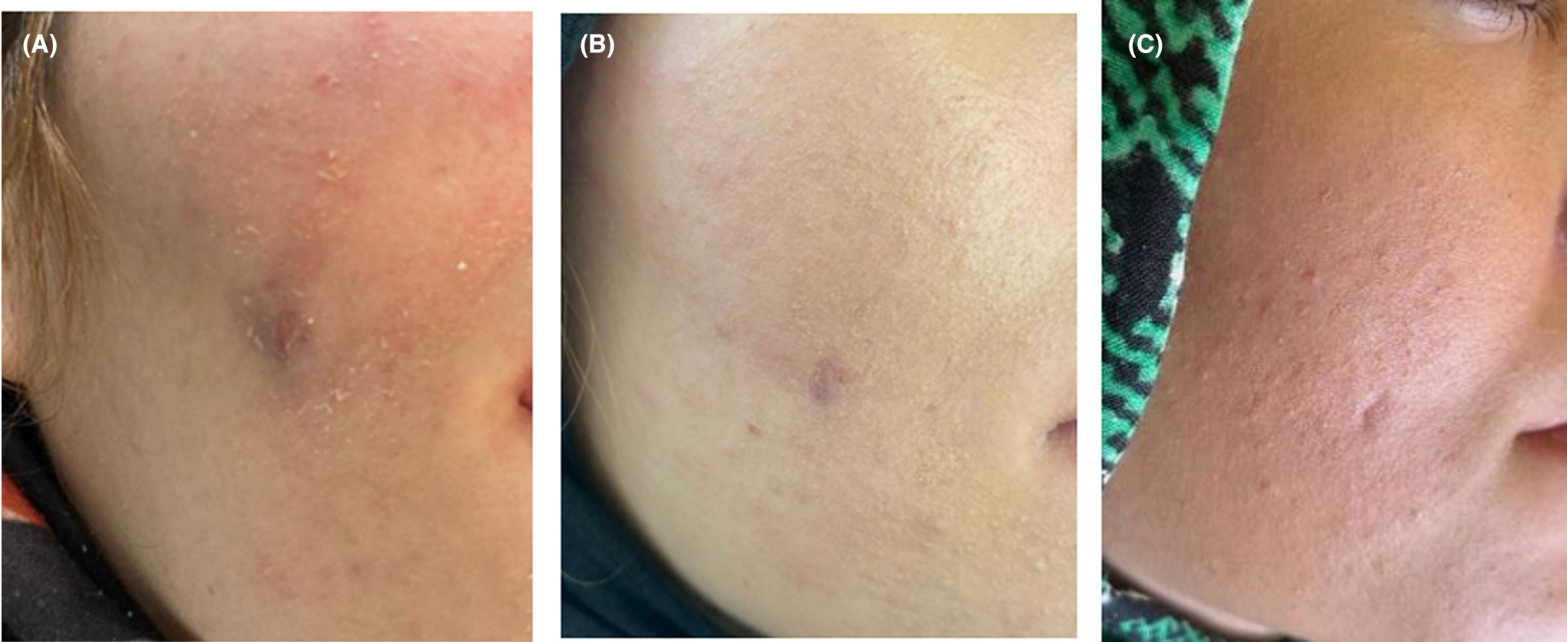
Follow‐up photographs after 1 month (A), 4 months (B), and 12 months (C).

A panoramic radiograph was ordered which revealed mucosal thickening in the maxillary sinus adjacent to the palatal root of the maxillary first molar (Figure 2A). The tooth had undergone a poor‐quality endodontic treatment 4 years earlier (Figure 2B). The patient did not recall any history of abscess, pain, or tenderness after endodontic treatment. Despite no pain on palpation, a cord‐like tissue was palpable extending deeply from the mucogingival junction of the respective tooth through the cheek and towards the facial nodule. The clinical findings such as long lasting acne‐like bump on the face, unresponsive to the administered treatments, pus collection on needle aspiration together with the obtained dental history were strongly suggestive of an odontogenic origin; thus, non‐surgical root canal retreatment was initiated. At the first appointment, gutta percha was removed. The second mesiobuccal canal was located, the canals were prepared, and copiously irrigated with 5.25% NaOCl solution using a side‐vented 30‐guage irrigation needle (Figure 2). A creamy mixture of calcium hydroxide and saline was then delivered into the canal spaces. Cone‐beam computed tomography (CBCT) was ordered (Figure 3C–F) for further evaluation of the bone destruction pattern and to ensure the presence of any additional missed canals other than the second mesiobuccal canal. Two weeks later, the lesion partially subsided but active pus discharge was seen from the palatal canal. Thus, the canals were disinfected using passive ultrasonic activation of 5.25% NaOCl solution. Triple antibiotic paste (TAP), containing ciprofloxacin, metronidazole, and minocycline (1:1:1), was then applied as the intracanal medicament. After 1 week, the canal dressing was removed. The root canals were dried with paper points, and obturated with 0.02 tapered gutta‐percha points (Meta BioMed) and AH26 root canal sealer (Dentsply, Maillefer) using cold lateral compaction technique (Figure 4A). The tooth was permanently restored with an endocrown. At 1‐month follow‐up, significant healing of the cutaneous lesion was noted (Figure 5A). At 4 months recall, the lesion was totally replaced with scar tissue (Figure 5B) and was completely resolved after 1 year (Figure 5C).

## 3 DISCUSSION

A Cutaneous sinus tract in the head and neck region might be a clinical manifestation of several infectious diseases, tumors, or congenital malformations. Odontogenic sinus tracts, mycobacterial infections, actinomycosis, deep fungal infections, and osteomyelitis are the most probable infectious sources. Some forms of acne lesions are also associated with cutaneous sinus tracts. Pyogenic granulomas, epidermal cysts, or squamous cell carcinomas are the possible tumoral diagnoses. Thyroglossal duct cysts, brachial cleft cysts, and salivary gland fistula have also been reported to be associated with such lesions.^4^

In the present case, intralesional steroid injection was first suggested by a dermatologist under the suspicion of an acne lesion. To rule out, it might be kept in mind that severe acne lesions resembling as observed in the present study, usually do not occur as a single lesion; whilst they are multiple nodulocystic lesions that appear mainly on the facial skin and may also occur on the upper arms, trunk, and back.^5^

Epidermoid cyst, also known as the sebaceous cyst, is a non‐tender encapsulated subepidermal keratin‐filled nodule which is more likely to appear on the face, neck, and trunk.^6^ In the present case, the lesion was a tender nodule and only pus was collected in needle aspiration; thus, epidermoid cyst was not a probable diagnosis.

Infected parotid gland sinus or fistula was another differential diagnosis. Salivary gland fistulas develop due to trauma, surgery, or infections. Similarly, parotid gland infections may cause a fistula primarily due to trauma, actinomycosis, tuberculosis, syphilis, salivary calculi, or malignancies. As the parotid duct crosses the outer surface of the masseter muscle and lies close to the skin, therefore is vulnerable to injury.^7^ In the present case, the patient did not report any history of trauma or surgery in the area. In clinical examination, on milking of the parotid gland, ejection of saliva was normal with a free flow and normal color, and the patient did not report any pain.

Chronic odontogenic infections can form intra/extra‐oral sinus tracts, that is, a communication between the root canal system and gingival or skin surface, respectively.^8^ Formation of whether an intra‐ or extra‐oral sinus tract following an odontogenic infection basically depends on the tooth type, position of tooth apices, virulence of bacteria, decreased immunity of the host, and low resistance of the adjacent structures.^9^ Patients with cutaneous sinus tracts are more likely to be initially visited by a dermatologist or a plastic surgeon; thus, such lesions tend to be misdiagnosed and mismanaged, particularly when the location of the lesion is atypical.

Odontogenic cutaneous sinus tracts are prevalently associated with mandibular teeth; thus, they commonly appear on the chin and the lower jaw.^10^ However, there are published reports of cutaneous odontogenic sinus tracts on the cheek,^2^, ^11^ inner canthus of the eye,^12^ intranasal areas,^13^, ^14^ and nasolabial fold.^10^, ^11^, ^15^ When a maxillary first molar's buccal apices are located beyond the buccinator muscle attachments, the odontogenic sinus tract may appear on the skin. In physical examination, a palpable cord‐like tissue connecting the cutaneous lesion to the bone surface adjacent to the roots' apices was found in the present case, which has been mentioned by several authors as a distinctive clinical finding.^1^, ^2^ Unlike the present case, dimpling is a common finding in such cases.

Odontogenic cutaneous sinus tracts tend to recur unless the main source of infection is eliminated through root canal treatment or tooth extraction.^1^, ^2^, ^11^, ^15^ Our patient also reported occasional antibiotic therapy, which would result in temporary shrinkage of the lesion; however, the lesion would recur soon after discontinuation of antibiotic therapy.

In the present case, maxillary sinus mucosal thickening adjacent to the palatal root of the first molar, which was primarily found on the panoramic radiograph, along with poor‐quality endodontic treatment of the respective tooth, albeit asymptomatic, suggested the presence of a chronic periapical infection which could result in either an intra‐ or extra‐oral odontogenic sinus tract. On CBCT images, periapical lesions associated with the first maxillary molar root apices were observed, and the buccal cortical plate adjacent to the mesiobuccal root was perforated by the expansion of the lesion. These findings strongly suggested a relationship between the suspected tooth and the cutaneous lesion.

After making the definite diagnosis, a clinician may decide to save the tooth by an endodontic intervention, via non‐surgical root canal treatment or retreatment. Alternatively, the clinician may decide to extract the suspected tooth, if the tooth is unrestorable. In the present case, due to the restorability of the tooth and the patient's preference in saving the tooth over extraction, non‐surgical root canal retreatment was performed. Fortunately, at the follow‐up sessions favorable response to the treatment was revealed.

Active pus discharge from the apical region of the palatal root canal at the second session was noticed. Thus, sodium hypochlorite solution was agitated using passive ultrasonic activation technique, and a more potent intracanal medicament (TAP) was delivered into the canal spaces. Several previous studies have highlighted the role of sodium hypochlorite activation in intratubular and intracanal microbial load reduction.^16^, ^17^, ^18^ TAP, which has been reported to outperform modified TAP, double antibiotic paste (DAP), and calcium hydroxide mixtures in terms of antimicrobial efficacy, were applied as inter‐appointment intracanal medicaments in previous studies.^19^, ^20^ This strategy was proven to be effective; since in the present study no active discharge was observed at the final appointment and the canals were thoroughly obturated.

Despite impossibility of sinus tract tracing due to the distance between the lesion and the suspected tooth; the pattern of bone destruction on CBCT images, failure of the previous treatment plans, and the onset of the healing process of cutaneous lesion during the treatment sessions, supported our clinical diagnosis and indicated the direct relation of the suspected tooth and the lesion. Although short‐term treatment outcomes were favorable, long‐term evaluation is required to ensure treatment success.

## 4 CONCLUSION

The present case report highlights the necessity of thorough extra‐ and intra‐oral examinations before any intervention. It also highlights the role of multidisciplinary approach and background knowledge in management of cutaneous sinus tracts.

## AUTHOR CONTRIBUTIONS

**Pegah Sarraf:** Conceptualization; methodology; supervision. **Mehrfam Khoshkhounejad:** Conceptualization; investigation; writing – review and editing. **Maryam Babaahmadi:** Methodology; validation; writing – original draft; writing – review and editing. **Fatemeh Hamidzadeh:** Methodology; writing – original draft.

## FUNDING INFORMATION

This research received no specific grant from any funding agency in the public, commercial, or not‐for‐profit sectors.

## CONFLICT OF INTEREST STATEMENT

The authors deny any conflict of interest.

## ETHICS STATEMENT

For clinical cases, the local ethics committee considers that the patient's consent is sufficient.

## CONSENT

Written informed consent was obtained from the patient to publish this report in accordance with the journal's patient consent policy.

## Data Availability

The data supporting the findings of the present study, are available from corresponding author upon request.
